# Targeted Therapy for Restoring CFTR Activity: From Experimental to Clinical Features

**DOI:** 10.3390/ijms27156919

**Published:** 2026-08-01

**Authors:** Sara Allushi, Mariarita Virgulti, Giovanna Blaconà, Giampiero Ferraguti, Adriana Eramo, Marco Lucarelli

**Affiliations:** 1 Department of Experimental Medicine, Sapienza University of Rome, 00161 Rome, Italy; sara.allushi@uniroma1.it (S.A.); mariarita.virgulti@uniroma1.it (M.V.); giovanna.blacona@uniroma1.it (G.B.); 2 Department of Oncology and Molecular Medicine, Italian Institute of Health, 00161 Rome, Italy; adriana.eramo@iss.it; 3 Pasteur Institute Cenci Bolognetti Foundation, Sapienza University of Rome, 00161 Rome, Italy

**Keywords:** cystic fibrosis, CFTR, targeted therapy, modulators, epigenetics

## Abstract

Cystic fibrosis (CF) is one of the most common rare genetic diseases. It is caused by pathogenic variants of the cystic fibrosis transmembrane conductance regulator (CFTR) gene. More than 2200 variants have been identified in the *CFTR* gene that need detailed knowledge and functional characterization in order to develop specific therapeutic strategies. The traditional therapy for CF relied on addressing symptoms through mucolytic and antibiotic treatments, respiratory physiotherapy and aerosol therapy. However, in the last few years, the development of small new molecules targeting and restoring the underlying CFTR channel defect marked an important step in CF treatment. In addition, the implementation of patient-specific cellular models allowed the evaluation of pharmacological responses, leading to therapeutic advances in the direction of personalized treatment. The focus of this review is to describe and discuss the strategies for restoring the CFTR functional defects depending on the specific *CFTR* pathogenic variants. In particular, the review highlights the possible application of experimental and clinical drugs to CF treatment, which may allow improvements in patients’ quality of life and life expectancy. The in vitro testing of therapeutic drugs (theratyping) is performed nowadays through the use of several cellular models, especially those derived from patient-specific tissues. This topic is a hot point in CF research and the review also aims to provide an overview of the state of the art in theratyping. The novelty of this review is the integrated view of the most recent achievements in precision diagnostics and therapy of CF at the molecular, cellular and clinical level, which are able to change the natural history of this disease.

## 1. Introduction and Search Strategy

Cystic fibrosis (CF) is an autosomal recessive genetic disorder due to pathogenic variants of the cystic fibrosis transmembrane conductance regulator (CFTR) gene, located in the long arm of chromosome 7 (7q31.2) [1]. The corresponding wild-type protein has 1480 amino acids and, although classified as a member of the ATP-binding cassette transporter family, it works as a cAMP-dependent channel permeable to chloride and bicarbonate anions. CFTR is mainly expressed on the apical membrane of epithelial cells characterizing different tissues, and it consists of two transmembrane domains, TMD1 and TMD2, two nucleotide binding domains, NBD1 and NBD2, and a regulatory domain (R) organized from the N- to C-terminus as follows: N-TMD1-NBD1-R-TMD2-NBD2-C [2].

The disease is characterized by various clinical conditions, as functional defects of CFTR compromise different organs depending on its tissue location and function [3]. The first condition is the actual CF, denoted by poly-symptomatic forms affecting multiple organs, with the main pathological consequence in the lungs, where altered chloride transport leads to a reduction in physiological airway surface liquid hydration [4]. This osmotic unbalance also results from the inability of mutated CFTR to regulate epithelial Na+ channel (ENaC) activity [4]. Therefore, the formation of a very thick and viscous mucus leads to impaired ciliary transport in the airway, causing frequent lung infections, inflammation and bronchiectasis. In addition, defective CFTR affects the gastrointestinal system with the production of viscous bile at hepatic level and the obstruction of pancreatic ducts not allowing the release of the respective juices [4,5]. The second clinical condition is characterized by an oligo- or mono-symptomatic disease defined as CFTR-related disorders (CFTR-RD), which widely differ in terms of severity [6]. The third most recently described category is the CF screening positive inconclusive diagnosis (CFSPID) represented by infants who tested positive at newborn screening but do not fully align with diagnostic criteria [7]. Finally, the obstructive azoospermia is the mildest mono-symptomatic CF form, noted as congenital bilateral atresia of vas deferens (CBAVD), because of their greater susceptibility to dehydration than other tissues during development [8].

In sweat glands, CFTR impairment is responsible for very salty sweat because both CFTR and ENaC channels decrease reabsorption activity [9]. This characteristic of people with CF (pwCF) was already known, as it dates back to an ancient folklore that roughly said: “Woe to the child whose forehead tastes salty when kissed, he is cursed and will soon die” [10]. Since sweat chloride concentration is higher in pwCF compared to healthy subjects, Gibson and Cooke have developed a diagnostic biochemical assay for CF based on this feature [11].

CF is one of the most prevalent rare genetic diseases with an incidence ranging from 1/3000 to 1/6000 live births in European countries [12]. Globally, more than 150,000 people are estimated to be living with CF across 94 countries, while approximately only 65% of them have received a certain diagnosis [13].

CF is a complex disease and pwCF exhibit different clinical manifestations based on the severity of *CFTR* pathogenic variants. As the residual functionality level of CFTR decreases, further clinical symptoms will occur in various anatomical regions, such as the sweat glands, lungs and, finally, the pancreas [14,15]. Indeed, the pancreatic insufficiency is considered an index of disease severity, not closely related to the clinical injury, rather because this symptom occurrence suggests that the CFTR functional levels are extremely low [14,15]. Instead, the lung damage is the most severe clinical manifestation in pwCF that could led to a fatal respiratory failure [15].

The wide clinical spectrum and variability in disease severity observed in CF largely reflect its genetic heterogeneity and the subsequent impact on CFTR protein function. The remarkable progress achieved over recent decades in CFTR biology has profoundly changed the clinical management of the disease. The unique contribution of this review consists of how a novel and integrated view of the most recent achievements in CF about CFTR molecular genetics, targeted therapy, patient-specific models, theratyping strategies and future experimental platforms can further change the natural history of the disease. This growing and integrated understanding is leading towards a real personalized medicine of CF.

A comprehensive literature search was independently conducted by two investigators (S.A. and M.V.) using the PubMed database to identify relevant articles published through May 2026. Search terms included “CFTR modulator”, “targeted therapy”, “patient-derived models”, “theratyping” and “clinical trial”. Additional information was obtained from other sources, including the https://clinicaltrials.gov/ and the Drug Development Pipeline of the Cystic Fibrosis Foundation to identify ongoing and recently completed clinical trials, as well as DrugBank and MedChemExpress to retrieve information on the chemical structures and the International Non-proprietary Name (INN) of the compound discussed in the review.

## 2. *CFTR* Pathogenic Variant Classification

To date, 2282 variants have been identified in the *CFTR* gene (The Human Gene Mutation Database, HGMD, last accessed 5 July 2026) and initially grouped into six different mutational classes based on the defect type of CFTR. Class I is characterized by variants that lead to no *CFTR* transcription (I subtype A), representing a novel category with the more severe phenotype unrescuable by corrective therapy, or to no protein production (I subtype B), such as premature termination codons (PTCs), large deletions or severe splicing pathogenic variants [16]. Examples are G542X (legacy name for this pathogenic variant and the others below), which is the second most frequent *CFTR* pathogenic variant in pwCF [17], and W1282X. The most prevalent CF-causing variant belongs to class II and consists of the deletion of three nucleotides in the exon 11 encoding for the phenylalanine at position 508 of CFTR protein (F508del). F508del and the overall class II pathogenic variants lead to an abnormal protein folding and trafficking, resulting in premature CFTR degradation. Class III pathogenic variants, including G551D, cause a defective regulation of CFTR channel gating, while class IV variants result in a reduced conductance and consequent decrease in channel activity. Class V includes mainly the pathogenic variants that produce anomalous pre-mRNA splicing, affecting the amount of wild-type *CFTR* mRNA, and those affecting the *CFTR* promoter, reducing the transcription of *CFTR* mRNA, both with decreased functional protein synthesis. Lastly, the class VI group of pathogenic variants cause CFTR plasma membrane instability [18].

However, the same pathogenic variant can lead to multiple biochemical defects of CFTR, such as, for example, affecting both cellular trafficking and channel functionality. Moreover, pwCF often exhibit different pathogenic variants in trans on the two alleles of the *CFTR* gene, thus defining a compound heterozygous genetic condition, and some others are characterized by complex alleles with two or more *CFTR* variants even in cis. This genetic complexity has required a re-classification of *CFTR* pathogenic variants with an overlap among the different mutational classes [19]. The detailed molecular characterization of *CFTR* pathogenic variants, as part of a precision diagnostics approach, has become pivotal for precision medicine in CF, providing the biological basis for the current targeted therapies and for the development of personalized therapeutic strategies.

## 3. Cystic Fibrosis Precision Therapies

**Figure 1:**
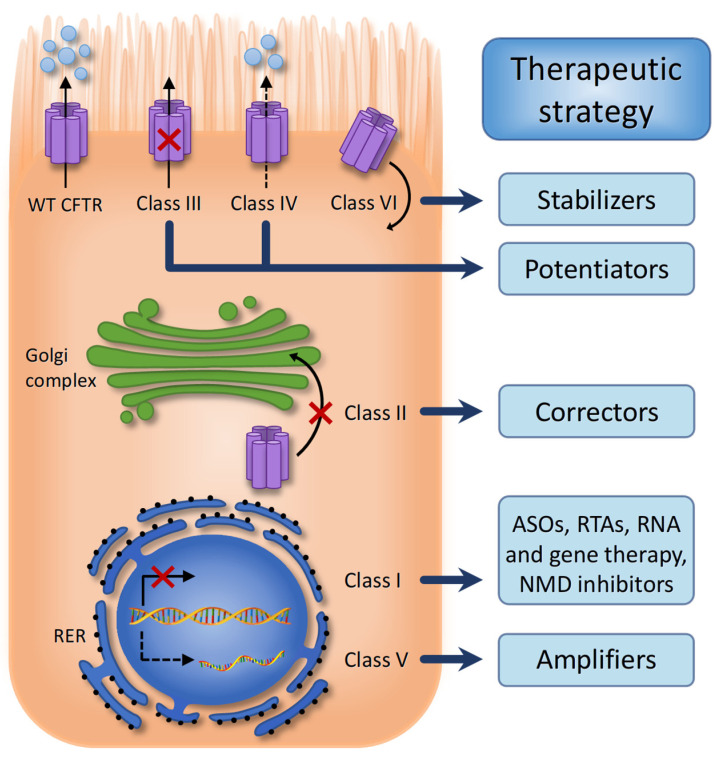
Specific therapeutic strategies to be employed depending on the CFTR functional defect. Class I variants could be targeted by readthrough agents (RTAs), antisense oligonucleotides (ASOs) and/or nonsense-mediated decay (NMD) inhibitor molecules, as well as by RNA and gene therapy. Class II variants are restored by corrector drugs, and class III and class IV by potentiators ones. Class V pathogenic variants could be targeted by amplifiers and class VI by stabilizers. The dashed arrows refer to a reduction, while the cross symbol on solid arrows denotes an interruption or a blockage. RER = rough endoplasmatic reticulum; WT = wild type.

In recent years there has been a major development in the search for targeted and effective CF therapies, especially as a result of the commercialization of CFTR modulator drugs. Targeted therapy requires detailed knowledge of *CFTR* pathogenic variants, as well as of CFTR functional defects. In particular, the classification of *CFTR* variants according to their pathogenic molecular mechanism allowed the development of specific therapeutic strategies to rescue the functionality of mutated CFTR (Figure 1).

### 3.1. Targeting Class I CFTR Variants

The class I nonsense pathogenic variants, which at best lead to the production of non-functional truncated protein, could be targeted using therapeutic approaches based on readthrough agents (RTAs), antisense oligonucleotides (ASOs), nonsense-mediated decay (NMD) inhibitors or gene therapy strategies.

Genetic editing represents a noteworthy approach to CF treatment aiming to correct the CFTR defect at the DNA level, with promising results at preclinical level [20,21,22]. However, there are numerous obstacles in implementing this therapeutic strategy, like the immune response to viral and non-viral vectors or the difficulties in reaching all target cells in the airway epithelium.

The delivery system can be based on non-infectious vehicles, such as lipid particles, or on viral vectors that exploit the natural infective proprieties of several viruses deprived of their pathogenicity. Currently, in the CF drug development pipeline, 4D-710, ARCT-032 and RCT2100 are gene therapy approaches using lipid nanoparticles or adeno-associated virus vectors that are under phase II clinical investigation (NCT05248230, NCT06747858 and NCT06237335, respectively). Although non-viral vectors are generally considered safer, they exhibit challenges in reaching the lung and delivering the genetic material into cells. The viral vector can overcome this limit, allowing higher transfection efficiency and long-term expression. The development of a third-generation self-inactivating lentiviral rSIV.F/HN vector, pseudotyped with Sendai virus F and HN envelope proteins in order to specifically target the apical surface of airway epithelia and allow orally inhaled administration, could represent a promising strategy in CF gene therapy [23]. Preclinical studies of this therapeutic approach (BI 3720931) have shown positive results in terms of efficacy and safety [24], and a phase I/II clinical trial is planned to end in 2026 (NCT06962852 and NCT06515002) [25,26].

On the other side, PTC124 (also known as Ataluren) and ELX-02 (also known as Exaluren) have been the most promising RTAs for the treatment of CF. The mechanism of action of these small molecules is to bypass the premature termination of the translation process by favoring the incorporation of near-cognate amino acids, allowing the production of a full-length CFTR. Although some initial clinical studies had demonstrated the efficacy of Ataluren [27,28], subsequent results failed to demonstrate significant improvements in pulmonary functionality between the treated and placebo group and the clinical studies on this molecule had been interrupted [29]. ELX-02 is a more recently developed molecule optimized to be safer and selective for premature PTCs in order to reduce off-target effects. The preclinical data of ELX-02 showed promising results in terms of efficacy [30,31] but the clinical evidence has not yet achieved sufficiently robust outcomes (NCT04135495 and NCT04126473).

A particular *CFTR* pathogenic variant that introduces a premature stop codon into the mRNA sequence is the single nucleotide substitution 3849+10kbC>T, historically categorized as class V for causing aberrant splicing. In order to prevent the abnormal inclusion of 84 intronic nucleotides in the *CFTR* mRNA sequence, an inhaled ASO drug named SPL84 was specifically designed. This RNA-based drug successfully completed a phase I clinical trial, demonstrating its safety and good tolerability [32,33,34], and a phase II clinical trial is now underway to test the preliminary efficacy in adult pwCF carrying at least one copy of the aforementioned splicing pathogenic variant (NCT06429176).

It is known that the introduction of a PTC induces the activation of the NMD process, a surveillance cellular mechanism involved in the degradation of abnormal transcripts [35]. Therefore, another possible strategy to rescue CFTR functionality is to use inhibitors of the NMD process, aiming to increase the *CFTR* mRNA quantity and consequently providing a greater amount of substrate that could be targeted by other therapeutic molecules. This approach of disrupting aberrant mRNA degradation may also be effective for those nonsense pathogenic variants that occur at the final coding region of the *CFTR* transcript, such as the rare W1282X, and thus would not significantly affect protein folding, allowing the production of a partially truncated CFTR. The suppressor of morphogenesis in genitalia 1 (SMG1) kinase is a key enzyme involved in the activation of this surveillance process, making it the optimal target to inhibit [36]. Several SMG1 inhibitor (SMG1i) molecules have been developed with this aim, and among them the most promising appears to be the compound 11j, which has been shown to specifically inhibit hSMG1 kinase activity in vitro [37]. However, further studies failed to demonstrate the in vivo therapeutic potential of SMG1i-11j due to its handling difficulties, thus requiring improvements before clinical application. To overcome this limit, new SMG1i compounds were identified through a high-throughput screening (HTS) approach and the KVS0001 molecule was demonstrated to be a specific inhibitor of SMG1 with adequate biophysical features for in vivo administration [38]. In order to interfere with the NMD process, different inhibitory approaches targeting other proteins or factors involved in this cellular mechanism have been evaluated [39,40]. Indeed, several compounds enacting by mechanisms of action other than SMG1 inhibition have shown to efficiently suppress NMD activation; among these, Compound C is of great interest. This molecule, known to inhibit specific protein kinases, has also been shown to disrupt NMD activity through the reduction of specific NMD factor levels, including SMG5, SMG6 and the helicase UPF1, representing a starting point for future investigations [41].

A drug that was investigated as a therapeutic approach for class I *CFTR* pathogenic variants is Amlexanox, which has been clinically approved for decades as an anti-inflammatory and anti-allergic medication. Preclinical studies demonstrated the ability of this compound to stabilize PTC-containing mRNAs, leading to an increase in the transcript amount, and to induce the translation of full-length proteins by promoting readthrough [42]. This drug has been demonstrated to induce a stronger response compared to other readthrough agents, such as G418 (Geneticin) and PTC124, tested alone and in combination [42]. The dual efficacy of Amlexanox in promoting NMD inhibition and readthrough induction has also been evidenced in vitro by studying an inherited peripheral neuropathy, specifically the Charcot–Marie–Tooth disease, with a 10% estimated prevalence of nonsense genetic variations [43].

In this context, experimental approaches combining inhibition of the NMD process with therapeutic molecules like RTAs have already been studied for CF treatment, specifically in a G542X CF murine model. Indeed, experiments performed on mice-derived intestinal organoids and airway air–liquid interface (ALI) cultures revealed a greater synergistic efficacy of co-treatment with SMG1i-11j plus G418 compared to other treatment combinations with Amlexanox, PTC124 and some non-aminoglycoside readthrough agents [44]. However, G418 is highly toxic in vivo and despite various attempts to improve the safety profile, this aminoglycoside readthrough agent seems to be unsuitable for clinical application [45].

### 3.2. Targeting Class II, III and IV CFTR Variants

The life expectancy of pwCF has considerably improved in recent years, not only because of earlier diagnosis, but especially as a result of pharmacological therapy with CFTR modulators. Thanks to the HTS process, researchers were able to identify potential molecules capable of restoring the functionality of defective CFTR [46]. Among these, the most promising compounds Ivacaftor (IVA, VX-770), Lumacaftor (LUM, VX-809), Tezacaftor (TEZ, VX-661) and Elexacaftor (ELE, VX-445) were further characterized through preclinical and clinical studies [47,48,49,50,51].

**Figure 2:**
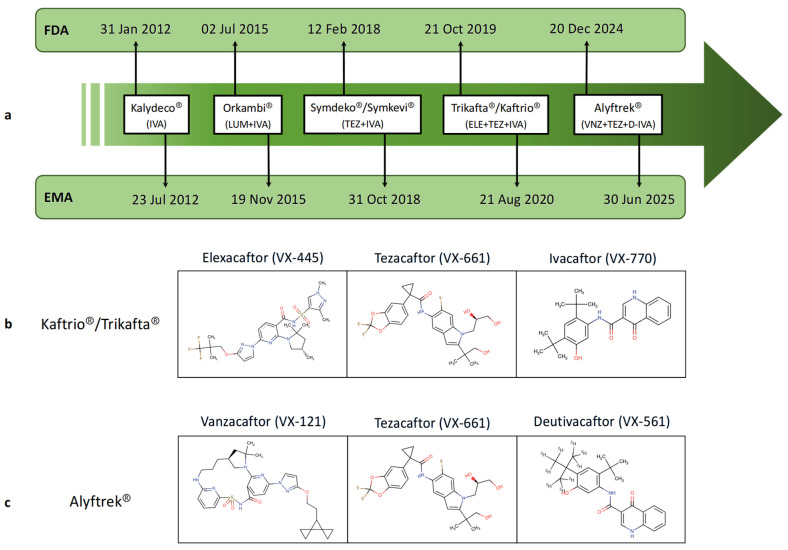
Graphic representation of the approved medications used in CF. (**a**) Timeline of the different FDA and EMA approvals for the five CFTR medications currently in clinical use for CF treatment. (**b**) Representation of the chemical structures of the compounds included in the triple combination Kaftrio^®^/Trikafta^®^. (**c**) Representation of the chemical structures of the compounds included in the triple combination Alyftrek^®^. FDA = Food and Drug Administration; EMA = European Medicines Agency; IVA = Ivacaftor; LUM = Lumacaftor; TEZ = Tezacaftor; ELE = Elexacaftor; VNZ = Vanzacaftor; D-IVA = Deutivacaftor. The structural formulas are from the free website DrugBank (https://go.drugbank.com/drugs, last accessed 5 July 2026).

To date, five CFTR medications are available for clinical use (Figure 2a).

The first one is the potentiator IVA, approved in 2012 by both the Food and Drug Administration (FDA) and the European Medicines Agency (EMA) (Figure 2a). This small-molecule drug specifically targets the gating impairment in terms of dysfunctional regulation and/or decreased conductance of CFTR (class III and class IV pathogenic variants), increasing channel opening [52]. Initially, IVA was approved only for pwCF ≥ 6 years old with at least one G551D pathogenic variant, in which the amino acid glycine is replaced by aspartate at position 551 in the NBD1 of the CFTR [53,54]. Over the years, the effectiveness of IVA has also been demonstrated for CFTR gating-affecting variants other than G551D [55,56], and currently it is approved in Europe for pwCF aged one month and older carrying the following *CFTR* pathogenic variants: G551D, G1244E, G1349D, G178R, G551S, S1251N, S1255P, S549N, S549R or R117H [57,58]. This means that IVA is the first and only CFTR modulator approved for CF treatment in such young individuals [59]. However, this small-molecule drug showed no clinical benefits in pwCF with the F508del homozygous genotype [60].

The deletion of phenylalanine in position 508, which is the most frequent pathogenic variant in the CF population, causes the misfolding of CFTR and consequent retention in the endoplasmic reticulum (class II pathogenic variants). Therefore, the F508del CFTR is degraded and cannot properly reach the plasma membrane of epithelial cells. In this regard, the second CFTR modulator drug, clinically approved in 2015, is a combination of the corrector LUM and the potentiator IVA (Figure 2a). Corrector drugs aim to rescue the CFTR misfolding and to enhance its delivery to the apical surface of epithelial cells. This pharmacological double combination marked an important step forward in the treatment of CF, as it was the first drug targeting the F508del pathogenic variant. At the beginning, LUM+IVA was prescribed to pwCF ≥ 12 years old carrying two copies of the F508del pathogenic variant [61]. Further clinical studies demonstrated the efficacy and safety of this drug combination even in children younger than 12 years [62,63,64,65], and in 2022 it was approved for the treatment of homozygous F508del pwCF aged 1 year and older [66]. However, LUM combined with IVA is proven to be ineffective in compound heterozygous pwCF with only one F508del allele [67] and an observational study showed side effects and drug-related intolerances [68], requiring further CF treatment improvements.

In 2018 the FDA, and a few months later the EMA, approved the commercialization of the third CFTR modulator drug characterized by the combination of TEZ and IVA (Figure 2a). TEZ is a corrector drug with a similar chemical structure to LUM, as both share the same binding site on the CFTR. These molecules belong to the first-generation corrector drugs and act by inserting into a deep pocket of TMD1, mainly through van der Waals interactions, stabilizing the mutated CFTR [69,70]. The combined treatment TEZ+IVA has proven to be effective with a more favorable side effect profile and less pharmacological interference [71,72]. This achievement allowed an expansion of the CF population that can benefit from CFTR modulator treatment, targeting not only F508del homozygous pwCF but also compound heterozygous ones with different responsive *CFTR* pathogenic variants on the other allele. Currently in Europe, the co-treatment TEZ+IVA is available in clinical use for pwCF aged 6 years and older who are F508del homozygous or F508del compound heterozygous with at least one of the following *CFTR* pathogenic variants: P67L, R117C, L206W, R352Q, A455E, D579G, 711+3A>G, S945L, S977F, R1070W, D1152H, 2789+5G>A, 3272-26A>G, 3849+10kbC>T [73].

A further CFTR modulator developed is ELE, a second-generation corrector that, unlike the first-generation ones, specifically binds to a hydrophobic site on the TMD2 of CFTR [74]. The ETI combination consists of ELE and TEZ correctors, targeting misfolding, and the IVA potentiator, enhancing gate opening, and it represents the fourth drug approved in 2019 by the FDA and a year later by the EMA (Figure 2a,b). This crucial achievement was supported by several clinical trials demonstrating pulmonary function improvements and a reduction in lung exacerbations and respiratory symptoms [49,75,76,77,78]. In particular, the synergistic efficacy in restoring the maturation and the functionality of the defective CFTR was obtained through the complementary mechanism of action of the two different types of correctors [79]. The triple combination was initially approved only for pwCF carrying at least one F508del allele but, thanks to preclinical data demonstrating the efficacy of CFTR modulators on a well-established in vitro cellular model, in December 2020 the FDA approved the extension of ETI’s use to additional responsive 177 rare non-F508del *CFTR* variants (https://investors.vrtx.com/news-releases/news-release-details/vertex-announces-fda-approvals-trikaftar, last accessed 5 July 2026). After exactly four years, the FDA expanded the therapeutic indication to further responsive 94 ultra-rare *CFTR* variants other than F508del (https://news.vrtx.com/news-releases/news-release-details/vertex-announces-us-fda-approval-trikafta, last accessed 5 July 2026). Recently, the expanded label for ETI in Europe has allowed its inclusion for pwCF with at least one non-class I pathogenic variant (https://news.vrtx.com/news-releases/news-release-details/european-commission-approves-expanded-label-kaftrior-combination, last accessed 5 July 2026). Therefore, the clinical approval and the expanded eligibility of ETI in pwCF aged 2 years and older is currently bringing significant benefits and radically changing pwCF’s quality of life [80,81,82,83].

A new triple therapy (VTD) characterized by the combination of correctors Vanzacaftor (VNZ, VX-121) and TEZ plus the potentiator Deutivacaftor (D-IVA, VX-561) emerged at the end of 2024 in the CF treatment landscape (Figure 2a,c). Clinical trial data showed positive results in terms of efficacy and safety, demonstrating improved pulmonary function and a greater reduction in sweat chloride concentration compared to ETI treatment in pwCF older than 12 years [84,85]. The benefits of this novel treatment have also been shown in children aged between 6 and 11 years old [86], while trials on children aged 2 to 5 years are currently underway (NCT05422222 and NCT05844449). The innovative aspect of this triple combination is the possibility for it to be taken once a day, which greatly improves therapy adherence, bringing positive implications from a lifestyle perspective.

Therefore, the investigation of new molecules targeting the underlying CFTR defect could lead to additional therapeutic advances towards personalized treatment [87,88,89]. In this regard, VX-828 is a novel next-generation CFTR corrector developed to improve channel folding and trafficking that is currently under early clinical evaluation in combination with TEZ and Deutivacaftor (NCT06154447).

### 3.3. Targeting Class V and VI CFTR Variants

An emerging class of modulator drugs in CF therapy is “amplifiers”, compounds that act by increasing the *CFTR* mRNA expression levels [90,91]. The amplificatory strategy aims to provide a higher amount of *CFTR* transcripts, and consequently a higher amount of mutated protein, that could be corrected and/or potentiated by other modulators in a synergistic way [92]. On the other hand, pathogenic variants with some CFTR residual function could also benefit from this approach without the need for other modulators. Focusing on these goals, the amplificatory strategy may be useful not only in the presence of class V pathogenic variants, but also in the presence of pathogenic variants belonging to other mutational classes.

A novel category of amplifier molecules has been identified through the HTS approach [90]. PTI-CH was the initially selected compound that has shown in vitro to increase the *CFTR* mRNA levels and to significantly enhance the rescue of mutated CFTR when combined with the corrector VX-809 and the potentiator VX-770 [93]. These results suggested an important role for the amplifier as an adjuvant to potentiate a therapeutic response. The pharmacodynamic comprehension of this compound allowed the development of other candidates with similar structures and improved target affinity [92]. To date, the experimental compound Nesolicaftor (PTI-428) is the only CFTR amplifier that has undergone clinical investigations to assess its safety and effectiveness in pwCF, both alone and in combination with other CFTR modulators (NCT03258424, NCT02718495, NCT03500263, NCT03591094 and NCT06468527).

**Figure 3:**
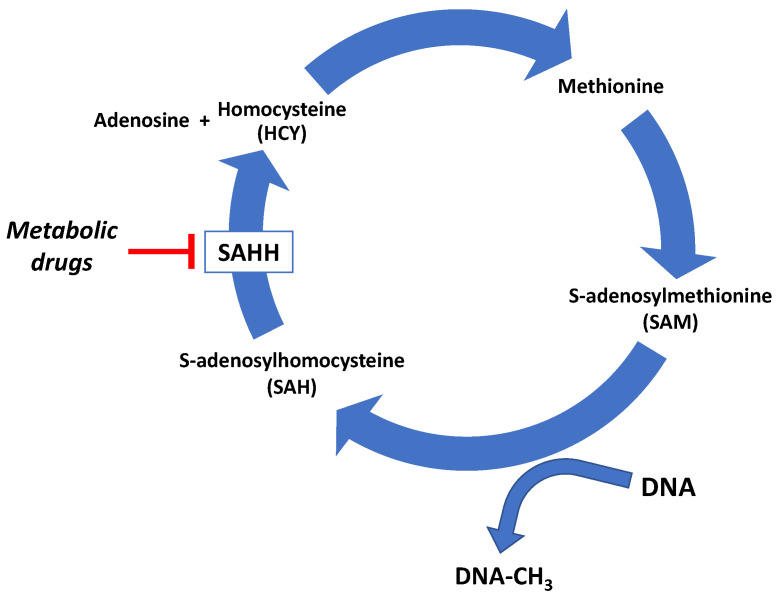
Schematic representation of the S-adenosylmethionine (SAM) cycle. Metabolic drugs exist that specifically inhibit the activity of S-adenosylhomocysteine hydrolase (SAHH) enzyme, leading to a reduction in SAM levels.

Epigenetics appears to be an attractive target for amplificatory approaches. Previous studies suggested that chromatin structure [94,95,96,97,98,99,100,101], DNA methylation patterns [94,102], and miRNAs [103,104] are involved in the modulation of the *CFTR* gene expression, although contrasting results have also been published [105,106]. Associations between altered DNA methylation patterns of the *CFTR* gene and cancer have also been evidenced [107,108,109,110]. Despite this evidence, little attention has been paid to therapeutic approaches for CF based on epigenetic interventions. Some data from the literature evidenced the possibility to act on histone deacetylase or DNA methyltransferase inhibitors for *CFTR* gene expression regulation [111]. However, the opportunity to act directly on DNA methylation patterns was nearly unexplored. In addition, the integration of a possible epigenetic action with the current modulatory therapy for CF has not yet been attempted. Starting from these poor experimental data, some working hypotheses can be formulated about a possible therapeutic epigenetic approach for CF. Since DNA methylation is generally associated with inhibition of gene expression [112], it is reasonable to suppose the possibility that a DNA hypomethylating agent could potentially increment transcript levels in the context of an amplificatory strategy for CF. This therapeutic approach could enter the preclinical stage by studying some compounds with a DNA hypomethylating effect. Some clinically approved DNA hypomethylating drugs already exist, such as 5-Azacytidine and Decitabine, which are mainly employed for the treatment of myelodysplastic syndrome and acute myeloid leukemia [113,114]. However, they appear inappropriate for CF treatment because they act by incorporating into DNA and irreversibly inhibiting the methylation process, thereby leading to cellular toxicity. Furthermore, this kind of drugs has the risk of non-specific effects on the epigenome and promotion of oncogenic processes. On the contrary, metabolic drugs, acting at the biochemical level on S-adenosylmethionine (SAM), which is the main cellular donor of the methyl group, could be a valid option as hypomethylating agents. These drugs interfere with the SAM cycle by inhibiting S-adenosylhomocysteine hydrolase activity, as demonstrated, also by us, in other contexts (Figure 3) [115,116,117,118]. The final effect is a reduction in SAM levels, limiting the supply of the methyl groups but allowing epigenetic regulation under cellular control, with fewer limitations and a lower risk of off-target effects [119]. However, there is a current lack of direct clinical validation in CF and extensive preclinical studies are required to assess efficacy, airway epithelium selectivity and safety.

Although the specific therapeutic approaches act on the underlying defect of the mutated CFTR, its rescued functionality could be undermined by the peripheral quality control (PQC) mechanism, a cellular process involved in the conformation-dependent ubiquitination of proteins at the plasma membrane level, leading to their internalization and degradation [120]. This typically occurs in CFTR proteins characterized by class VI pathogenic variants, resulting in a shorter half-life and lower apical surface expression. In this regard, the “stabilizers” act on the CFTR membrane instability by targeting the molecular components of the PQC system, aiming to reduce the degradation rate and to increase the surface permanence of the channel protein [120]. Cavosonstat (N91115) was the first CFTR stabilizer compound to undergo a phase II clinical trial investigation (NCT02589236, NCT02724527) [121], but unfortunately the drug development was interrupted due to insufficient results in terms of pulmonary function improvements. From a preclinical point of view, a promising approach to interfere with the PQC process and consequently stabilize the surface permanence of CFTR is to simultaneously inhibit the RFFL and RNF34 ubiquitin ligases, which are involved in the chaperone-independent ubiquitination and lysosomal degradation of CFTR [122,123]. This experimental strategy could potentially enhance the efficacy of ETI treatment in restoring CFTR activity by increasing protein membrane retention, with significant clinical implications.

All the mentioned compounds are summarized in Table 1.

## 4. Theratyping for pwCF Without Approved Therapeutic Options

While pwCF with the most frequent and well-characterized pathogenic variants have access to the approved CFTR modulator drugs, those with rare or unexplored variants are still orphan of modulatory therapies, mainly due to the difficulty of conducting experimental and clinical studies on a large population. Moreover, many *CFTR* pathogenic variants show multiple biochemical defects and belong to several mutational classes [19], resulting in a dramatic translational impact on the mutation-specific therapies.

“Theratyping” is an innovative approach aiming to personalize CF treatment according to the individual *CFTR* genotype. It is based on investigating the effects of modulators, either experimental or already approved in clinical use, in patient-specific cellular models [124,125]. This strategy could improve the management of pwCF through the evaluation of specific responding *CFTR* pathogenic genotypes. Thereby, the therapeutic decision relies on identifying the effective treatment that demonstrates the in vitro functional recovery of CFTR.

One of the major challenges for CF theratyping is to establish suitable patient-specific cellular models that can accurately recapitulate the three-dimensional morphology and functional characteristics of lungs in vitro, mainly due to the limited availability of CF individuals’ tissue and scarce efficiency of cell expansion which lead to insufficient supply of experimental material. Furthermore, the respiratory epithelium’s composition is highly complex, consisting of at least seven different epithelial cell types (and relative subtypes), such as ciliated, secretory, basal, club, neuroendocrine and tuft cells, as well as the rare pulmonary ionocytes [126]. The latter is of particular interest since it is characterized by elevated transcript levels of *CFTR* and Forkhead Box I1, a marker of epithelial differentiation. Although representing only a small fraction of the airway epithelial cells, ionocytes appear to be important contributors to CFTR-mediated ion transport [127,128]. Preserving this cellular diversity is essential for testing drug efficacy in a personalized way and predicting the patient-specific response to therapies.

The technical difficulties of performing cellular models using primary cells are in vitro expansion and proper differentiation, since they rapidly undergo senescence processes and transformation into squamous cells. To overcome these limitations, stem-like reprogramming strategies have been developed. Specifically, the transient expression of four transcription factors (c-Myc, Oct3/4, Sox2 and Klf4) reprogrammed adult fibroblasts into induced pluripotent stem cells (iPSCs), which resemble embryonic stem cells in terms of morphology and pluripotency [129]. However, pluripotent stem cells exhibit a distinctive antigenic profile that can influence their interaction with the immune system [130], and the use of retroviral vectors for reprogramming has been associated with genomic instability and a higher risk of tumorigenicity [131].

The Conditionally Reprogrammed Cells (CRC) methodology overcomes the iPSC limitations, supporting the exponential growth and long-term expansion of airway epithelial stem-like cells by using irradiated mouse fibroblasts as a feeder layer and the Rho-kinase (ROCK) inhibitor Y-27632 [132,133]. ROCK1 and ROCK2 are downstream effector proteins of the Rho pathway that phosphorylate different types of substrates on serine and threonine residues. This phosphorylation influences several cellular processes such as apoptosis, cellular adhesion and actin stabilization [134]. The synthetic compound Y-27632 inhibits the kinase activity of the ROCK protein by competing with ATP for binding to the catalytic site [135]. Therefore, cultures of human primary cells on irradiated fibroblasts in the presence of Y-27632 express several epithelial stem cell markers such as CD44, ΔNp63α, and integrins α6 and β1, with a decrease in Notch signaling and an increase in nuclear β-catenin levels [136]. The first studies on CRCs were conducted on keratinocytes, and their karyotype characterization revealed no differences between immortalized and primary cells [134]. Subsequently, other types of human tissues were cultured by CRC methodology [132]. The reprogramming culture conditions allow the obtainment of adult stem-like cells, preserving not only their differentiation potential but their physiological functions too [133]; after the removal of CRC conditions, cells differentiate in the original tissue, restoring the expression of differentiative markers [137]. The possibility to obtain through the CRC methodology long-term stable cultures of airway epithelial stem cells (AESCs) derived from lung explants, bronchial biopsies and nasal brushings of pwCF with different pathogenic variants (CF-CRC-AESC) represents a powerful tool for therapeutic studies [138,139]. The collection of airway epithelial cells by nasal brushing represents a minimally invasive methodology, allowing the sampling of a wide range of different mutated genotypes and the possibility to repeat it more than once [140]. The CRC methodology has proved to be particularly effective, as almost 100% of samples taken from pwCF resulted in long-term cell culture [139].

Another strategy to induce primary cell expansion is the “dual-SMAD inhibition” (SMADi), involving the use of small molecules inhibiting the transforming growth factor-β (TGF-β) and the bone morphogenetic protein (BMP) signaling pathways, which are both Sma- and Mad-related protein (SMAD)-dependent [141].

To induce mature respiratory differentiation, the expanded primary cells can be cultured in the ALI condition using a transwell support, with the basolateral cell compartment in contact with the medium and the apical cell surface exposed to the air, mimicking the in vivo airway situation and allowing the formation of a pseudostratified epithelium characterized by tight junctions, functional beating cilia as well as mucus secretion [139]. The lineage-specific markers such as acetylated α-tubulin and mucin 5B are expressed after 4 weeks of differentiation [139]. While both CRC and SMADi approaches seem to be useful to expand nasal epithelial cells, CRC-ALI cultures exhibit higher cilia beating frequency than SMADi-ALI cultures [142]. This suggests that CF-CRC-AESC cultures constitute an experimental model highly suitable to represent at best the characteristics of pwCF. Therefore, ALI cultures are good candidates for pharmacological tests that can be conducted both for CF individuals with a genotype that guarantees access to treatment and for those whose genotype does not have access to that drug yet [139]. However, 2D cell cultures lack the extracellular matrix (ECM), which is involved in the formation of cellular networks of epithelial cells in vivo.

In addition, 3D cell cultures are grown in a matrix or by developing a scaffold that mimics the ECM. Several protocols have been developed to generate human airway organoids from different cell sources, including basal and secretory cells, as well as iPSCs [143]. Airway organoids derived from lung biopsies or bronchoalveolar lavage cells, cultured in medium integrated with biochemical factors such as R-spondin 1, fibroblast growth factors and TGF-β inhibitors, result in a polarized epithelium constituted by basal cells, functional ciliated cells and secretory cells [144,145]. Another powerful approach to produce airway organoids is based on the use of nasal brushing cells. In particular, the brushing-derived AESCs obtained through the CRC methodology could be cultured with a growth-factor-reduced matrigel, generating in about 21 days 3D organoid structures with the apical membrane facing inward and characterized by a pseudostratified epithelium with motile cilia visible in the lumen [139,146].

Intestinal epithelial cells represent an additional and valuable resource for studying the relationship between the *CFTR* pathogenic variants and the in vitro cellular response. These cells can be obtained from rectal biopsies and used to generate patient-derived intestinal organoids. In particular, they arise from Lgrf5+ stem cells able to proliferate and differentiate into specialized cells of the intestinal epithelium, recapitulating structural and functional features of the tissue [147].

Therefore, nasal and intestinal 3D organoids have become a relevant model system for CF research, enabling the prediction of individual responses to CFTR modulatory therapies through a functional test based on the Forskolin-induced swelling (FIS) assay [148,149,150,151]. The comparison of drug treatment conducted on both intestinal and nasal organoids, obtained from the same CF individual, showed a concordance in response, demonstrating their validity for therapeutic in vitro evaluation [152]. The FIS assay involves the stimulation of the CFTR channel, the activation of which induces an influx of chloride and water into the lumen of the organoid, generating a change in size. The swelling is reduced or absent in organoids generated from cells of pwCF in respect to those generated from wild-type cells. The size variation of the organoids following FIS under therapeutic treatment depends on the effects of modulatory therapy on the specific *CFTR* mutated genotypes [145]. There is growing evidence that the results obtained from the FIS assay following treatment with modulatory therapies are predictive of a patient-specific response [139,146,153]. It has also been observed that there is a correlation between the swelling of the organoid and the improvement in lung function [154]. Nasal, bronchial and rectal epithelia have all been found to be particularly useful for the functional analysis of CFTR and for the development of a personalized therapy [149].

Despite the pivotal role that 2D cell cultures and 3D organoids play in the theratyping of CF, further improvements are needed in accurately reproducing the complexity of human lung tissue. Even organoids fail to reconstruct the structural and mechanical characteristics of the organs. Cell cultures and organoids are not able to recreate the tissue–tissue interface between the microvascular endothelium and parenchyma where the transport of all necessary elements occurs [155]. Although it is not yet possible to fully reconstruct the physiological environments and the functionality of the organs, thanks to engineering techniques it has been possible to develop chips which can reproduce a physiologically relevant microenvironment. These chips have been used to develop lung, brain, kidney, intestinal, and gastric models and to reproduce blood vessels [155].

Rather than recreating the entire organ, the primary goal is to reproduce small functional units that are able to recapitulate its activities. The simplest models consist of a single chamber characterized by a single cell type. More complex models, however, are made up of two or more micro-channels connected to each other by a porous membrane. For this type of model, different cell types are used to recreate the interfaces between tissues [156]. The organ-on-a-chip methodology can imitate the physiological environment of human organs with the capacity to regulate key parameters. These models are made of four key components: living cell tissues, sensing, microfluidics and stimulation or drug delivery. The microfluidic component is important for driving the cells to their location and in balancing the incoming and the outgoing fluids in the culture process [157]. The complexity of the human lung is demonstrated by the high number of different cell types in it [158], but also by the structure of the alveoli, responsible for gas exchange, which is particularly complicated. However, thanks to the fluid flow and sustained gas exchange, this high challenging model can also be established with microfluidics [159]. On the other hand, it should be considered that the lung is a very dynamic organ subjected to various mechanical stresses and pressures exerted by both blood and air [160].

Lung-on-a-chip models represent an innovative bioengineering technology that more faithfully recapitulate airway physiology, allowing a better simulation of the biological microenvironment. A lung-on-a-chip model, characterized by both fluidic and pneumatic components and able to reproduce the movements of the alveolar barrier during breathing, has been developed using cells isolated from human lung surgical resection [159]. The chip is made of polydimethylsiloxane and contains two channels separated by an ECM membrane. One channel is lined with alveolar epithelial cells, while the other is lined with a vascular endothelium, mimicking the physiological alveolar–capillary interface for gas exchange. Culture medium is perfused through the endothelial channel, while the epithelial channel is exposed to the air, simulating ALI conditions [161]. Moreover, small airway-on-a-chip platforms have also been developed to support the differentiation of a pseudostratified and mucociliary bronchiolar epithelium [162]. These systems may be useful tools for investigating respiratory diseases and, in this context, a human airway-on-a-chip model for CF characterized by a differentiated pseudostratified respiratory epithelium has been developed [163]. The airway chips derived from pwCF showed increased mucus accumulation and higher ciliary beat frequency compared to those obtained from healthy individuals, demonstrating their ability to more closely recreate the features of CF pulmonary disease [163]. Furthermore, pwCF typically experience chronic lung inflammation, and these CF airway chips have shown an increased level of pro-inflammatory cytokines compared to healthy individuals, providing further evidence of the chips’ capability to mimic in vivo conditions [163]. Thus, the dynamic microenvironment, the multicellularity (endothelial, epithelial and immune cells) and the cellular crosstalk allow lung-on-a-chip to recreate a more faithfully complexity of the CF physiopathology compared to the 3D organoid cultures [164]. This aspect is particularly advantageous for predicting the in vitro pharmacological response of modulatory therapies in a personalized manner. For example, compassionate programs, such as the French one [165,166], providing clinical evidence of the beneficial effects of ETI therapy in pwCF subgroups carrying undruggable variants, could help to validate the lung-on-a-chip platform as a predictive experimental tool in CF theratyping. Similar to the other 2D or 3D approaches, a crucial aspect of lung-on-a-chip is the experimental evaluation of the CFTR residual and recovered function. To this end, recently developed nanoelectrodes that are able to measure chloride concentration [167] could be suitably integrated. The lung-on-a-chip model could offer future advantages and could represent the basis for the development of patient-specific therapies in the field of CF.

All mentioned experimental models used in CF are summarized in Table 2.

## 5. Conclusions and Future Prospects

The landscape of CF treatment has undergone a paradigm shift, transitioning from purely symptomatic management to a sophisticated era of precision medicine. Although pwCF’s life expectancy and quality of life have significantly improved in recent years thanks to the development of CFTR modulators, there are continuous efforts to identify new or repositioned drugs in order to expand therapeutic options. This is mandatory for pwCF with rare and/or undruggable genotypes. In addition, complexity to treatment strategies is heightened considering that the same pathogenic variant can lead to multiple biochemical defects. Beside the aim of directly targeting the underlying biochemical defect of the CFTR, novel therapeutic approaches pointing in a wider manner to cellular pathways can also be considered, for example the targeting of cellular mechanisms involved in the transcriptional rate, nonsense-mediated decay or epigenetic regulation. With this aim, the concept of theratyping should be expanded to include not only the testing of CFTR-targeted drugs, but also strategies acting on whole pathways, possibly in a synergic way. Beyond drug testing and the development of precision therapies, attention should also be directed to the overall CFTR cellular functions, possibly impaired as a consequence of the mutated *CFTR* genotype. The systematic functional characterization of mutated *CFTR* genotypes has not been attempted yet, although it is crucial for the amelioration of precision diagnostics and the genotype–phenotype relationship.

The targeted characterization and personalized therapeutic approaches may be achieved thanks to the implementation of patient-specific cellular models, which allows genotype-specific evaluation of the basal function and pharmacological response within the individual genetic background. Cellular systems are already available, no matter how sophisticated they are at present, but are far from a full recapitulation of the high complexity of the respiratory epithelium. As a future direction, an effort should be made towards the production of integrated devices with growing cellular and engineering complexity that are able to simulate, as fully as possible and in a dynamic manner, the complex biological and mechanical functions of the lung environment. With this aim, a method for assessing CFTR function, which can be continuously evaluated over time in an easy and cost-effective manner, is crucial. Nanoelectrodes and lung-on-a-chip appear to be particularly promising as integrated cellular devices.

While CFTR modulators have changed the clinical history of CF, the integration of advanced patient-specific models with innovative methods for the dynamic evaluation of CFTR and functions of the respiratory epithelium are now essential to extend these benefits to every individual, moving toward truly personalized therapeutic management.

## Figures and Tables

**Table 1:** Summary of approved and investigational therapies for CF, including their mechanism of action and International Non-property Name (INN). The reported development stage corresponds to the highest phase reached by each compound, including discontinued clinical programs. Readers are referred to the main text for details on current status of clinical trials. ASO = antisense oligonucleotides; RTA = readthrough agents; NMD = nonsense-mediated decay; TMD1 = Transmembrane domain 1.

Mutational Class	CFTR Defect	Therapeutic Strategy	Compound	INN	Mechanism of Action	Development Stage
**Class I variants**	No protein production	Gene therapy	4D-710	-	Adeno-associated viral vectors designed to deliver a healthy *CFTR* gene to lung cells	Clinical
			BI 3720931	-	Third-generation lentiviral vector pseudo-typed with Sendai virus F and HN envelope proteins to target the apical surface of airway epithelia	Clinical
		mRNA therapy	ARCT-032	-	Lipid-mediated delivery system of a full-length *CFTR* mRNA to lung cells	Clinical
			RCT2100	-		
		ASOs	SPL84	-	Short antisense oligonucleotide designed to bind the 84-nt intronic sequence in the *CFTR* pre-mRNA, preventing its aberrant inclusion in the mature mRNA	Clinical
		RTAs	PTC124	Ataluren	Promotes the readthrough of premature termination codons by favoring the incorporation of near-cognate amino acids, allowing the production of a full-length CFTR	Clinical
			ELX-02	Exaluren		Clinical
			-	Amlexanox		Preclinical *
		NMD inhibitors			Inhibition of key enzymes involved in the activation of the NMD surveillance process to promote *CFTR* mRNA stability	
			SMG1i-11j KVS0001 Compound C	-		Preclinical
**Class II variants**	Abnormal protein folding and trafficking	Correctors	VX-809	Lumacaftor	First-generation correctors acting by insertion into a hydrophobic pocket of TMD1, stabilizing CFTR folding and trafficking	Approved
			VX-661	Tezacaftor		Approved
			VX-445	Elexacaftor	Next-generation correctors that enhance CFTR folding and intracellular trafficking through complementary binding sites	Approved
			VX-121	Vanzacaftor		Approved
			VX-828	-	Corrector that promotes CFTR folding and trafficking	Clinical
**Class** **III–** **IV variants**	Dysfunctional channel gating-conductance	Potentiators	VX-770	Ivacaftor	Increases the CFTR open probability by stabilizing the open channel conformation	Approved
			VX-561	Deutivacaftor	Deuterated analog of Ivacaftor with the same mechanism of action but a prolonged half-life and improved pharmacokinetic properties	Approved
**Class V variants**	Reduced mRNA transcription	Amplifiers	SMG1i-11j KVS0001 Compound C	-	Inhibition of key enzymes involved in the activation of the NMD surveillance process to promote *CFTR* mRNA stability	Preclinical
			PTI-428	Nesolicaftor	Improvement in *CFTR* mRNA’s stability and translation through association with specific RNA-binding proteins	Clinical
**Class VI variants**	Protein plasma membrane instability	Stabilizers	N91115	Cavosonstat	Promotion of the S-nitrosylation of proteins involved in CFTR quality control, thus limiting its ubiquitination and degradation	Clinical

* Amlexanox is a clinically approved drug, but in experimental/preclinical development for cystic fibrosis.

**Table 2:** Comparison of the different experimental models used in CF.

Experimental Models	Source and Cell Type	Physiological Relevance	Primary Application in CF	Main Advantages	Major Limitations
**Induced Pluripotent Stem Cells (iPSCs)**	Somatic cells reprogrammed into iPSCs	Ability to generate multiple differentiated cell types	- Structural characterization of rare *CFTR* variants - Bioengineering through CRISPR/Cas-mediated gene editing	- Virtually unlimited self-renewing cell source - Minimally invasive sampling	- Risk of genomic instability and tumorigenicity induced by reprogramming
**Conditionally Reprogrammed Cells of Airway Epithelial Stem Cells (CF-CRC-AESC)**	Primary airway epithelial cells expanded with irradiated feeder fibroblast and ROCK inhibitor (Y-27632)	Maintain stem-like condition of patient-derived airway cells; capable of airway differentiation	- Biobanking of patient-specific airway epithelial cells and generation of differentiated airway models	- Wide and rapid expansion - Biobank generation	- Require feeder cells - CFTR functional assays require subsequent differentiation
**Dual SMAD Inhibition Cultures (SMADi)**	Primary airway epithelial cells expanded by dual inhibition of TGF-β and BMP signaling	Maintain stem-like condition of patient-derived airway cells; capable of airway differentiation	- Biobanking of patient-specific airway epithelial cells and generation of differentiated airway models	- Feeder-free expansion - Biobank generation	- Moderate proliferative rate - Moderate in vitro differentiation efficiency - CFTR functional assays require subsequent differentiation
**Air–Liquid Interface Cultures (ALI)**	Airway epithelial cells derived from iPSCs, bronchial biopsy or nasal brushing	Recapitulates a pseudostratified, ciliated and mucus-secreting respiratory epithelium	- Functional characterization of rare *CFTR* variants - Preclinical testing of CFTR modulators - Evaluation of CFTR activity by functional assays	- High efficiency to reproduce physiological airway epithelium - Good candidates for pharmacological tests	- Lack of ECM - Low cell culture protocols (28-day differentiation)
**Airway 3D Organoids**	Airway epithelial cells derived from iPSCs, bronchial biopsy or nasal brushing	Three-dimensional model of the airway epithelium, recapitulating tissue architecture and cellular functions	- Functional characterization of rare *CFTR* variants - Preclinical testing of CFTR modulators - Evaluation of CFTR activity by functional assays	- Enable 3D tissue organization with ECM support - Good candidates for pharmacological tests - Strict correlation with clinical outcomes	- Functional testing through FIS assay requires highly specialized laboratory experts
**Intestinal 3D Organoids**	Primary Lgrf5+ stem cells isolated from rectal or intestinal biopsy	Three-dimensional model of the intestinal epithelium, recapitulating tissue architecture and cellular functions	- Functional characterization of rare *CFTR* variants - Preclinical testing of CFTR modulators - Evaluation of CFTR activity by functional assays	- Enable 3D tissue organization with ECM support - Good candidates for pharmacological tests - Correlation with functional response of airway organoids	- Moderately invasive sampling - Functional testing through FIS assay requires highly specialized laboratory experts
**Airway-on-a-Chip**	Airway epithelial cells derived from lung explant or bronchial biopsy	Enables tissue–tissue interactions and recapitulates key environmental factors	- Modeling CF airway pathophysiology under dynamic conditions - Evaluation of CFTR activity by functional assays	- Able to reproduce complex structures to study tissue–tissue interactions and cellular crosstalk	- High costs and manufacturing complexity - Require highly interdisciplinary expertise (bioengineering/biology)

This table is intended as a concise conceptual summary highlighting the key features, advantages and limitations of each experimental model for CF precision medicine applications.

## Data Availability

No new data were created or analyzed in this study. Data sharing is not applicable to this article.

## Abbreviations

The following abbreviations are used in this manuscript:

AESC	Airway epithelial stem cell
ALI	Air–liquid interface
ASO	Antisense oligonucleotide
BMP	Bone morphogenetic protein
CBAVD	Congenital bilateral atresia of vas deferens
CF	Cystic fibrosis
CFSPID	Cystic fibrosis screening positive inconclusive diagnosis
CFTR	Cystic fibrosis transmembrane conductance regulator
CFTR-RD	CFTR-related disorders
CRC	Conditionally reprogrammed cells
D-IVA	Deutivacaftor
ECM	Extracellular matrix
ELE	Elexacaftor
EMA	European Medicines Agency
ENaC	Epithelial Na+ channel
ETI	Elexacaftor + Tezacaftor + Ivacaftor
FDA	Food and Drug Administration
FIS	Forskolin-induced swelling
HTS	High-throughput screening
iPSC	Induced pluripotent stem cell
IVA	Ivacaftor
LUM	Lumacaftor
NBD	Nucleotide binding domain
NMD	Nonsense-mediated decay
PQC	Peripheral quality control
PTC	Premature termination codon
pwCF	People with cystic fibrosis
ROCK	Rho-kinase
RTA	Read-through agent
SAM	S-adenosylmethionine
SMAD	Sma- and Mad-related protein
SMADi	Dual-SMAD inhibition
SMG1	Suppressor of morphogenesis in genitalia 1
SMG1i	SMG1 inhibitor
SMG1i-11j	SMG1 inhibitor, compound 11j
TEZ	Tezacaftor
TGF-β	Transforming growth factor-β
TMD	Transmembrane domain
VNZ	Vanzacaftor
VTD	Vanzacaftor + Tezacaftor + Deutivacaftor
